# pH-Responsive Dual-Network PVA Films Integrating CNC-Stabilized *Mosla chinensis* Essential Oil Emulsions for Active Food Packaging

**DOI:** 10.3390/foods15132401

**Published:** 2026-07-07

**Authors:** Huiqiong Wu, Yuxuan Zhu, Huan Liu, Yingying Deng, Zhipeng Wang, Hongning Liu, Zhe Li, Liangshan Ming

**Affiliations:** Key Laboratory of Modern Preparation of TCM, Institute for Advanced Study, Ministry of Education, Jiangxi University of Chinese Medicine, Nanchang 330004, China; wuhuiqiong1@jxutcm.edu.cn (H.W.); zhuyuxuan@jxutcm.edu.cn (Y.Z.); liuhuan14@jxutcm.edu.cn (H.L.); dengyingying1@jxutcm.edu.cn (Y.D.); wangzhipeng@jxutcm.edu.cn (Z.W.); 19820002@jxutcm.edu.cn (H.L.)

**Keywords:** *Mosla chinensis* essential oil, tannic acid-Fe^3+^ complex, pH-responsive, active film, yam preservation

## Abstract

This study developed a pH-responsive dual-network polyvinyl alcohol (PVA) active packaging film by integrating cellulose nanocrystal (CNC)-stabilized *Mosla chinensis* essential oil (EO) emulsions with a tannic acid–Fe^3+^ metal–phenolic network (MPN). The CNC-stabilized emulsion improved EO dispersion and retention in the PVA matrix and contributed to network reinforcement through hydrogen-bonding interactions, while the dynamic MPN further strengthened the film structure and acted as a pH-gated domain for regulating EO release. Composite film achieved high UVA and UVB blocking efficiencies of 90.06% and 99.60%, respectively, together with improved mechanical, barrier, antioxidant, and antibacterial properties. Compared with neutral conditions (pH = 7), EO release increased by 179.9% and 181.3% at pH = 4 and pH = 9, respectively, confirming the pH-gated release behavior of the film. In yam preservation, composite film effectively delayed browning, weight loss, firmness decline, and spoilage. Metabolomic analysis further indicated that the film treatment regulated phenylpropanoid metabolism and flavonoid biosynthesis, which may contribute to maintaining antioxidant-related metabolic homeostasis. This work offers a pH-responsive controlled-release active packaging strategy for browning-prone fresh produce.

## 1. Introduction

**Figure 1:**
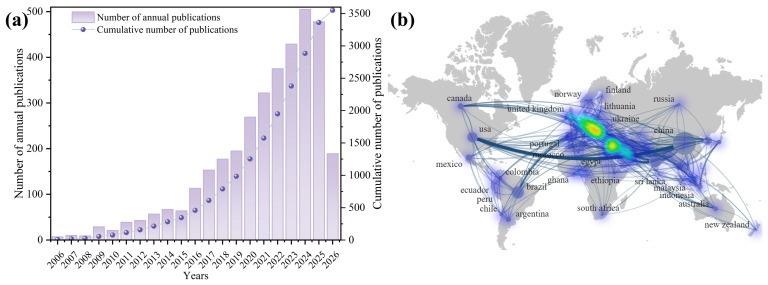
Insights from statistical analysis of literature on films. (**a**) The number of annual publications and the cumulative number of publications from Web of Science; (**b**) International collaboration network graph. In panel (**b**), the color gradient from blue to green/yellow indicates increasing collaboration density or activity, with warmer colors representing countries or regions with stronger publication or collaboration intensity. (Note: All literature was obtained from the Web of Science core collection with the search term TS = (“essential oil” OR “essential oils” OR “volatile oil” OR “volatile oils” OR “ethereal oil” OR “refined oil”) AND TS = (“film” OR “thin film” OR “antimicrobial film” OR “antioxidant film” OR “active packaging films” OR “UV-blocking films” OR “controlled release films”), and the search period is 1 January 2006–31 may 2026, and the article type is “article” and “review”, and the language is English. A total of 3551 articles were found.)

Essential oils are natural volatile mixtures rich in bioactive phytochemicals, such as phenolics, terpenes, and aldehydes, exhibiting broad-spectrum antimicrobial, antioxidant, and insecticidal activities [1]. Among these, *Mosla chinensis* essential oil (EO), characterized by its high carvacrol content, effectively inhibits postharvest pathogen growth, scavenges reactive oxygen species, and delays oxidative deterioration in fresh produce [2]. Owing to their natural origin, high efficiency at low doses, and absence of synthetic residues, EOs are increasingly regarded as sustainable alternatives to conventional chemical preservatives and a hot topic of research in the field of active food packaging in recent years (Figure 1). Despite these advantages, the practical application of EOs in hydrophilic polymer-based packaging films remains challenging. Their high volatility, strong hydrophobicity, and poor compatibility with polar matrices often lead to phase separation, rapid leaching, and premature loss of bioactivity during storage. This not only shortens the effective preservation window but may also cause sensory deviations due to excessive EO exposure, thereby limiting the reliability of such systems in real-world supply chains.

To address these limitations, various encapsulation strategies have been explored to improve EO stability and regulate release behavior, including emulsification [3], cyclodextrin inclusion [4], liposomes [5] and microcapsules [6]. Among these, emulsions stabilized by cellulose nanocrystals (CNCs) offer a robust platform for integrating hydrophobic EOs into hydrophilic film-forming matrices. CNCs, derived from abundant renewable biomass resources, possess amphiphilic properties and strong interfacial adsorption capacity due to their surface hydroxyl groups [7]. They anchor at the oil–water interface to form dense interfacial layers, thereby enhancing EO dispersion and preventing phase separation. Furthermore, CNCs can interact with polyvinyl alcohol (PVA) chains via hydrogen bonding to reinforce the film matrix [8]. Significantly, food deterioration triggers changes in the acid-base environment. However, simple static encapsulation alone primarily improves physical stability and passive retention, relying on free diffusion for release rather than enabling precise, stimulus-responsive delivery. Therefore, incorporating dynamic crosslinking networks is a promising route to achieve on-demand release.

Metal–polyphenol networks (MPNs) are a class of supramolecular network materials formed through self-assembly via coordination between metal ions (including Fe^3+^, Cu^2+^, etc.) and polyphenolic compounds. Shi et al. confirmed the safety of MPN through cytotoxicity and hemocompatibility experiments, demonstrating their suitability for use in food contact materials [9]. Among these, the MPN formed by the coordination of tannic acid (TA) with Fe^3+^ exhibit reversible bonding characteristics and can serve as molecular gates for constructing intelligent packaging systems [10]. TA, a natural polyphenol rich in galloyl groups and phenolic hydroxyls, forms hydrogen bonds with PVA and CNC, promoting a dense hydrogen-bonded network. Simultaneously, Fe^3+^ acts as a coordination center, forming a dynamic MPN structure with TA that enhances the mechanical strength, UV-barrier properties, and antioxidant capacity of biopolymer films [11]. Crucially, the coordination between TA and Fe^3+^ is reversible and pH-responsive, allowing the network to function as a dynamic gate that regulates EO diffusion in response to pH fluctuations associated with food spoilage. While most studies on MPN-based films have focused on static performance optimization or freshness indication, the use of MPNs as dynamic gates for controlled bioactive release remains limited. For example, Yang et al. developed a carboxymethyl chitosan (CMCS)/TA-Fe^3+^ hydrogel film in which reversible TA-Fe^3+^ coordination enables pH-responsive color changes, making it a visual freshness indicator in processed meat products [12]. Therefore, integrating CNC-stabilized EO emulsions with MPN is expected to concurrently improve EO dispersion, enhance film mechanics, and achieve pH-triggered release.

**Figure 2:**
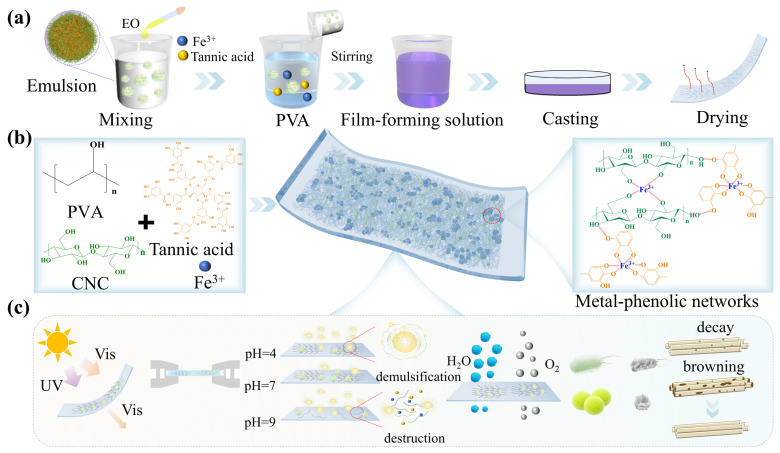
Schematic diagram of preparation, formation mechanism, and functionality of PVA-PE-TF film. (**a**) Preparation process of PVA-PE-TF film. (**b**) Mechanism of PVA-PE-TF film formation. (**c**) Functions and applications of PVA-PE-TF film.

This study developed a pH-responsive dual-network PVA active packaging film by integrating CNC-stabilized EO emulsions with MPNs (Figure 2). The CNC-stabilized emulsion was designed to enhance EO dispersion and retention within the hydrophilic PVA matrix, while the MPN functioned as a dynamic gating structure to regulate EO release under acidic or alkaline conditions. The structural interactions, physicochemical properties, antioxidant and antibacterial activities, and pH-responsive release behavior of the composite films were systematically characterized. Furthermore, fresh yam, a highly perishable tuber crop susceptible to enzymatic browning and microbial decay, was selected as a model food to evaluate the preservation efficacy of the optimized film [13]. Untargeted metabolomic analysis was conducted to elucidate the potential regulatory mechanisms of the packaging system on antioxidant-related metabolic pathways, with a focus on phenylpropanoid metabolism and flavonoid biosynthesis. This work provides a feasible strategy for developing intelligent active packaging systems that synergize emulsion-based EO delivery with MPN-mediated dynamic gating for the preservation of perishable agricultural products.

## 2. Materials and Methods

### 2.1. Materials

CNC were supplied by Nanjing Ruiniu Energy & Environmental Materials Technology (Nanjing, China), and their zeta potential, particle size, and X-ray diffraction (XRD) information are shown in Figure S1a–c. PVA (Mw: 89,000–98,000, 95%) was purchased from Rhawn (Shanghai, China). Tannic acid (TA) (≥98%), Tris (hydroxymethyl) aminomethane hydrochloride (≥99.9%), 2,2-diphenyl-1-picrylhydrazyl (DPPH, >97.0%), Levofloxacin (>98.0%) were obtained from Aladdin (Shanghai, China). FeCl_3_·6H_2_O (99%) and Tween 80 were sourced from Macklin (Shanghai, China). Riboflavin (98%) was supplied by YuanYe Biotechnology (Shanghai, China). Glycerol (≥99.0%) was supplied from Xilong Science (Shantou, China). The bacterial strains Escherichia coli (*E. coli*) (ATCC 25922) and *Staphylococcus aureus* (*S. aureus*) (ATCC 25923) were obtained from the American Type Culture Collection (ATCC, Manassas, VA, USA). Fresh yams were purchased from the local market.

The dried herb of *Mosla chinensis* was obtained from Jiangzhong TCM Pieces Co. (Nanchang, China), and its EO was isolated using a hydrodistillation method. The components of the essential oil were identified by GC-MS as described previously [14].

### 2.2. Preparation of CNC-Stabilized EO Emulsion

The CNC-stabilized EO emulsion was prepared by homogenizing a mixture of 1% (*w*/*v*) cellulose nanocrystals (CNC), 1% (*w*/*v*) Tween 80, and 10% (*w*/*v*) EO using an FA 25 high-speed shear (FLUKO Shanghai Equipment Co., Ltd., Shanghai, China) at 22,000 rpm for 20 min in an ice bath [2].

### 2.3. Characterization of CNC-Stabilized EO Emulsion

According to the method described by Wu et al., the zeta potential of the emulsion, which was diluted 40X in deionized water, was determined using the analyzer (Nano ZS, Malvern Instruments, Malvern, UK) employing the dynamic light scattering (DLS) technique [7]. The emulsion droplet size was measured via a laser diffraction particle size analyzer (Mastersizer 2000, Malvern Instruments, Malvern, UK). Prior to analysis, each sample was subjected to ultrasonic treatment for 1 min. The droplet morphology of the CNC-stabilized EO emulsion was examined using a digital camera (DS-Fi2, Nikon, Tokyo, Japan) integrated with NIS-Elements Imaging Software (version 4.60, Nikon, Tokyo, Japan). Overall, 10 μL of the emulsion was carefully placed on a glass slide, covered with a coverslip to avoid sample evaporation and contamination, and then visualized under an optical microscope at 40× magnification.

### 2.4. Preparation of Composite Films

The films were prepared using a solution-casting method [15]. Initially, PVA was dissolved in ultrapure water under magnetic stirring at 85 °C (200 rpm) until completely dissolved, yielding a 5% (*w*/*v*) PVA solution. Subsequently, 1.5% (*w*/*v*) glycerol was added as a plasticizer and mixed thoroughly. For the PVA-PE and PVA-PE-TF formulations, the CNC-stabilized EO emulsion was incorporated into the PVA/glycerol solution and stirred until homogeneous. For PVA-PE-TF I–V, TA solutions prepared in 10 mmol/L TRIS buffer (pH 8.0) and FeCl_3_ solutions were added as paired concentration levels. Table 1 shows the designations of all films and the composition of the TA, FeCl_3_, and emulsions they contain. Finally, each film-forming solution was stirred until homogeneous, poured into Petri dishes, and dried at 40 °C for 17 h.

### 2.5. Characterization of Composite Films

#### 2.5.1. Appearance, Thickness, Color and Optical Properties

The appearance of films was documented. The average thickness of each film was obtained by measuring ten randomly selected points using a digital spiral micrometer (Sanliang, Shanghai, China, accuracy 0.001 mm) [15]. The Commission Internationale de l’Eclairage (CIE) color space, a widely accepted system for standardized and precise color evaluation, was employed to assess the color characteristics of the samples. Color parameters were determined with a spectrophotometer (3nh, Shenzhen, China) [4]. The CIE system quantifies three colorimetric parameters: L* for brightness (0 = black, 100 = white); a* represents the red–green component (positive = red, negative = green), and b* reflects the yellow–blue axis (positive = yellow, negative = blue). For each sample, measurements were taken at three randomly selected locations to ensure representativeness. Film transmission in the UV–vis region was characterized with a UV–vis spectrophotometer (UV-2600, Shimadzu, Kyoto, Japan). Samples of various PVA-based films (3 cm × 1 cm) were prepared to enable light passage. A blank UV–quartz cuvette served as the reference. Spectral measurements were recorded across 200–800 nm. The UV-blocking percentages for UVA and UVB were determined based on Equations (1) and (2) [16].

UVA blocking (%) = 100 − ∫_320_^400^T(λ) × d(λ)/∫_320_^400^d(λ) × 100 (1)

UVB blocking (%) = 100 − ∫_280_^320^T(λ) × d(λ)/∫_280_^320^d(λ) × 100 (2)

where T(λ) is the mean transmittance of the films, d(λ) the bandwidth, and λ the wavelength.

The film samples were exposed to a 16 W UV light for 30 days. A UV–vis spectrophotometer (UV-2600, Shimadzu, Kyoto, Japan) was utilized to determine their transmittance within the 200–800 nm range at eight-day intervals [17].

#### 2.5.2. Chemical Structure

FT–IR spectra of the films were acquired on a Fourier transform infrared spectrometer (INVENIO, Bruker, Ettlingen, Germany) to identify molecular bonding in the matrix, over 4000–600 cm^−1^ with 2 cm^−1^ resolution and 32 scans per sample [18]. Distinct characteristic absorption peaks of Fe^3+^-TA complex were measured via a UV–vis spectrophotometer (UV-2600, Shimadzu, Kyoto, Japan). Raman spectral date was acquired via a 785 nm excitation laser equipped on the Raman spectrometer (PERS-ER1705, Perser Technology, Suzhou, China) [19]. The surface chemical states of film samples were analyzed by XPS (K-Alpha, Thermo Scientific, Waltham, MA, USA) with Al Kα radiation (hν = 1486.6 eV), 400 μm spot size, 50 eV pass energy, and 0.1 eV step size at 12 kV. Data processing was conducted using the Thermo Avantage software (version 6.9, Thermo Scientific, Waltham, MA, USA) [20].

#### 2.5.3. XRD

XRD patterns of samples were obtained by X-ray diffractometer (D8 Advance, Bruker AXS, Karlsruhe, Germany). Film samples were scanned with Cu-Kα radiation (40 kV accelerating voltage, 40 mA current) over 2θ = 5–60°.

#### 2.5.4. Thermogravimetric Analysis (TGA)

The thermal behavior of PVA, PVA-PE, and PVA-PE-TF films was investigated via a thermogravimetric analyzer (STA449F5, NETZSCH, Selb, Germany). 5 mg of the specimen was heated programmably from 30 °C to 600 °C in a 50 mL/min nitrogen flow, at a constant 10 °C/min heating rate. The derivative thermogravimetric (DTG) profile was obtained by differentiating the TGA values [21].

#### 2.5.5. Morphology Characterization

As described previously by [21], scanning electron microscopy (SEM) of the film samples were characterized using a scanning electron microscope (Quanta 250, FEI Company, Brno, Czech Republic). Before imaging, samples were cryofractured into fragments in liquid nitrogen. Fragments were then positioned horizontally and vertically on conductive gel to observe surface and cross-sectional morphologies. Gold sputter coating was applied under vacuum conditions to enhance surface conductivity. Observations were conducted at an acceleration voltage of 10 kV.

High-resolution images of composite film samples were acquired using atomic force microscope (Multimode Icon, Bruker, Santa Barbara, CA, USA) in tapping mode, scanning at 0.89 Hz over a 5 μm × 5 μm area. Film surface roughness was analyzed via Bruker Nanoscope Analysis 3.00 software (version 3.00, Bruker, Santa Barbara, CA, USA ) [5].

#### 2.5.6. Water Contact Angle (WCA), Water Vapor Permeability (WVP) and Oxygen Permeability (OP)

A water contact angle goniometer (OCA20, DataPhysics instruments, Filderstadt, Germany) was employed to measure the tangential angle between a water droplet and the film surface, thereby evaluating the hydrophobicity of the film. At 25 °C, 2 μL of deionized water was dispensed onto the film surface [22]. Measurements were taken at five different locations on each film to ensure accuracy and repeatability.

WVP of films was determined by placing 1 g of anhydrous calcium chloride (desiccant) into a 5 mL capless centrifuge tube, sealing with a 3 cm× 3 cm film, and storing the tube in a desiccator with supersaturated NaCl solution (75% RH) at 25 °C for 48 h. Weight change in each tube was recorded. WVP was calculated by Equation (3) [15].

WVP (g∙mm/h∙m^2^ kPa) = (Δm × d)/(A × Δt × Δp) (3)

where Δm is the tube weight difference (g), (d) is film thickness (mm), A is the exposed area of the film (m^2^), Δt is time interval (h), and Δp is water vapor pressure difference (kPa).

OP was determined by placing 4 g deoxidizer (iron powder, activated carbon, sodium chloride) in 5 mL centrifuge tubes, sealing with 3 cm × 3 cm films, and storing in a desiccator at 25 °C and 75% RH for 48 h. Tubes were reweighed after storage. OP was calculated by Equation (4) [23].

OP (g/m^2^ h) = Δm/(A × Δt) (4)

where Δm denotes the mass of O_2_ absorbed by the deoxidizer (g), A denotes the area of the film covering the mouth of the centrifuge tube (m^2^), and Δt denotes the equilibration time (h).

#### 2.5.7. Mechanical Properties

Mechanical properties of the films, encompassing stress–strain, tensile strength (TS), elongation at break (EAB) and Young’s modulus, were evaluated via a universal testing machine (5305H, SUNS, Shenzhen, China) with specifications of 10 mm gauge width, 20 mm gauge length, and 25 mm/min crosshead speed [24].

### 2.6. pH-Responsive Release Behavior

With slight modifications on the methods by Wu et al., the pH-responsive release capacity of EO from the films was assessed using a UV–vis spectrophotometer (UV-2600, Shimadzu, Kyoto, Japan) at a detection wavelength of 277 nm, and the UV-Vis absorption spectra were recorded to investigate the pH dependence of MPN in the 350–600 nm wavelength range [25]. 1.0 g film sample was immersed in 200 mL release medium (10% *v*/*v* ethanol), with pH adjusted to 4.0, 7.0, and 9.0 to simulate physiological conditions. Samples were incubated in a 37 °C shaking water bath. At preset time intervals, 3 mL medium was withdrawn and replaced with fresh medium of corresponding pH to keep volume constant. The concentration of EO released was quantified based on a previously established calibration curve correlating absorbance (A) to concentration (C): A = 0.0147C − 0.0292 (R^2^ = 0.9999). Furthermore, encapsulation efficiency (EE) of EO within films was calculated using Equation (5).

EE (%) = (Mass of encapsulated EO)/(Mass of total EO) × 100%(5)

### 2.7. Antioxidant and Antimicrobial Activities

The antioxidant capacity of the films was evaluated with slight adjustments based on the method by [26], A 20 mg film sample was weighed, dissolved in 10 mL absolute ethanol, and ultrasonication for 30 min. Subsequently, 2 mL of the supernatant was mixed with 1 mL of 0.1 mM DPPH (2, 2-diphenyl-1-picrylhydrazyl) solution in ethanol. The mixture was vortexed and incubated in the dark for 30 min. after which absorbance was recorded at 517 nm by a UV–vis spectrophotometer (UV-2600, Shimadzu, Kyoto, Japan). 2 mL of ethanol as the blank. The DPPH radical scavenging activity was quantified by Equation (6) [15].

DPPH radical scavenging activity (%) = (A_0_ − A)/A_0_ × 100% (6)

where A_0_ denotes the absorbance of the blank control, and A denotes the absorbance of the sample.

The antimicrobial activity of PVA-PE-TF films against *E. coli* and *S. aureus* was assessed [22]. Bacterial suspensions (1.0 × 10^8^ CFU/mL) were evenly spread on LB agar and incubated at 37 °C for 30 min. Circular film disks were placed onto the agar surface. A levofloxacin-loaded tablet (15 µL, 50 µg/mL) served as the positive control, and phosphate-buffered saline (PBS) (15 µL) as the blank. Plates incubated at 37 °C for 17 h were assessed by measuring the inhibition zone diameters.

### 2.8. Application of Composite Films in Yam Preservation and Metabolomic Analysis

Three yams with good appearance, no obvious damage, and uniform size were selected for the preservation study. The yams were wrapped with PVA-PE and PVA-P-TF I films, respectively. An untreated group was used as the blank control, while a commercial polyethylene (ethylene homopolymer) film was employed as the control group, with an oxygen permeability of 13,905 cm^3^/(m^2^·24 h·0.1 MPa), a carbon dioxide permeability of 57,090 cm^3^/(m^2^·24 h·0.1 MPa), and a water vapor permeability of 56 g/(m^2^·24 h). All samples were stored at room temperature (25–28 °C) under a relative humidity of 40–45% for 8 days.

Yam firmness was determined with a Texture analyzer (TA.XT plus, Stable Micro Systems, Godalming, UK) For each fruit, three penetration measurements were performed at a depth of 3 mm, with a test speed of 2 mm/s and a trigger force of 5 g [27]. To assess moisture loss, the weight of the yams was measured before the experiment (W_0_) and every 24 h after the experiment began (W_i_). The weight loss percentage was calculated by Equation (7) [28].

W_loss_ (%) = (W_0_ − W_i_)/W_0_ × 100% (7)

where W_0_ and W_i_ correspond to the initial and measured weights, respectively.

Colorimetric analysis of yams was performed at room temperature every two days using a spectrophotometer (3nh, Shenzhen, China) according to the CIELAB scale of the CIE color space system. Color parameters included L* (lightness, 0 = black, 100 = white), a* (red–green axis; positive = red, negative = green), and b* (yellow–blue axis; positive = yellow, negative = blue). RGB values of digital images of preserved yams were also analyzed over 8 days using an online LAB-to-RGB converter (https://products.aspose.app/svg/zh/color-converter/lab-to-rgb, accessed on 7 August 2025) [29].

In addition, the BI, serving as an indicator of browning intensity, was calculated using the following Equations (8) and (9).

BI = (100(x − 0.31))/0.172(8)

x = (a* + 1.75 L*)/(5.645 L* + a* − 3.012 b*)(9)

Another approach to determine the BI involved sampling yam specimens treated with preservatives at predetermined intervals. Each sample was mixed with deionized water (1:10 *w*/*w*), followed by homogenization and centrifugation (6000 rpm, 20 min), with slight modifications to the protocol described in reference [30]. The supernatant was diluted tenfold, and its absorbance at 420 nm (A_420_) measured with a UV–vis spectrophotometer (UV-2600, Shimadzu, Kyoto, Japan). The BI was calculated as A_420_ × 10, in accordance with previously reported methods [31].

For metabolomic analysis, samples were freeze-dried using a Freeze dryer (Scientz-100F, Scientz, Ningbo, China) and homogenized into a fine powder with tissue grinder (MM 400, Retsch, Haan, Germany) at 30 Hz for 1.5 min. A 50 mg portion was extracted with 1200 μL of pre-chilled (–20 °C) 70% methanol/water at 4 °C, vortexed for 30s every 30 min for six cycles, and centrifuged at 12,000 rpm for 3 min; the supernatant was filtered through a 0.22 μm membrane into autosampler vials for analysis [32].

Metabolite analysis was performed on a UPLC-MS/MS system (ExionLC AD + QTRAP® 6500+, SCIEX, Framingham, MA, USA) on an Agilent SB-C18 column (1.8 µm, 2.1 mm × 100 mm; Agilent Technologies, Santa Clara, CA, USA) with solvent A (ultrapure water + 0.1% formic acid) and solvent B (acetonitrile + 0.1% formic acid) at 0.35 mL/min, 40 °C, and 2 μL injection volume. Mass spectrometry employed an electrospray ionization (ESI) source at 500 °C, with ion spray voltages of +5500 V (positive) and –4500 V (negative) and gas pressures of 50, 60, and 25 psi for ion source gas 1 (GS1), ion source gas 2 (GS2), and curtain gas (CUR), respectively. Data were acquired in multiple reaction monitoring (MRM) mode with nitrogen collision gas, and optimal MRM transitions were determined by tuning declustering potential (DP) and collision energy (CE), applying time-segmented monitoring according to retention times.

### 2.9. Statistical Analysis

Each experiment was independently conducted at least three times, and the obtained data are presented as the mean ± standard deviation (*n* = 3). Statistical analysis was performed using one-way analysis of variance (ANOVA) with SPSS software (version 21.0, IBM, Armonk, NY, USA). *p* < 0.05 was considered to represent a statistically significant difference.

## 3. Results and Discussion

### 3.1. Characterization of CNC-Stabilized EO Emulsion Analysis

**Figure 3:**
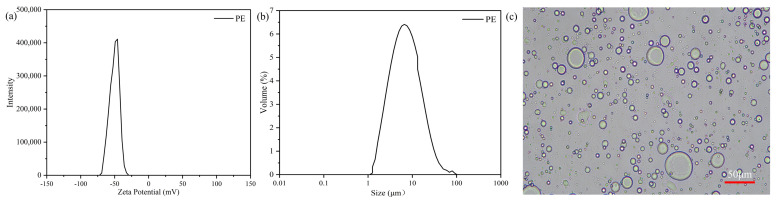
Characterization results of emulsion. (**a**) Zeta potential; (**b**) Particle size; (**c**) Microscopy at 40X.

Emulsion stability plays a crucial role in subsequent film-forming processes, and zeta potential and droplet size are critical parameters for evaluating emulsion stability. Typically, CNC formulations with absolute zeta potential values below 20 mV are considered unstable; values ranging from 20–30 mV indicate moderate stability; and values above 30 mV are generally regarded as highly stable [33]. The zeta potential of the emulsion was measured as −52.93 ± 1.89 mV, indicating that the emulsion droplets possess strong electrostatic repulsion, which effectively hinders their aggregation (Figure 3a). This phenomenon could be due to the adsorption of CNC at the droplet interface. The D_0.9_ value of the emulsion droplets was measured at 13.07 μm, with their particle size distribution shown in Figure 3b. Emulsion droplet size is largely governed by the thermodynamic energy of the system and the interface area, with smaller droplets contributing to reduced aggregation and improved stability of CNC-stabilized EO emulsion.

Figure 3c presents an optical microscopy image of freshly prepared CNC-stabilized EO emulsion, where smaller emulsion droplets are observed within the field of view. This observation indicates the successful formation of the emulsion system, which may be ascribed to the amphiphilicity of CNC, facilitating its localization at the O/W interface and the formation of a rigid particle film [34]. Furthermore, because CNC prepared with sulfuric acid possesses negatively charged functional groups, the strong electronegativity of these groups stabilizes the emulsion droplets through electrostatic repulsion and steric effects. This not only enhances the O/W interfacial area but also increases the participation of CNC particles during emulsification, thereby facilitating the formation of uniform emulsion droplets.

### 3.2. Characterization of Composite Films Analysis

#### 3.2.1. Appearance, Thickness, Color and Optical Properties Analysis

**Figure 4:**
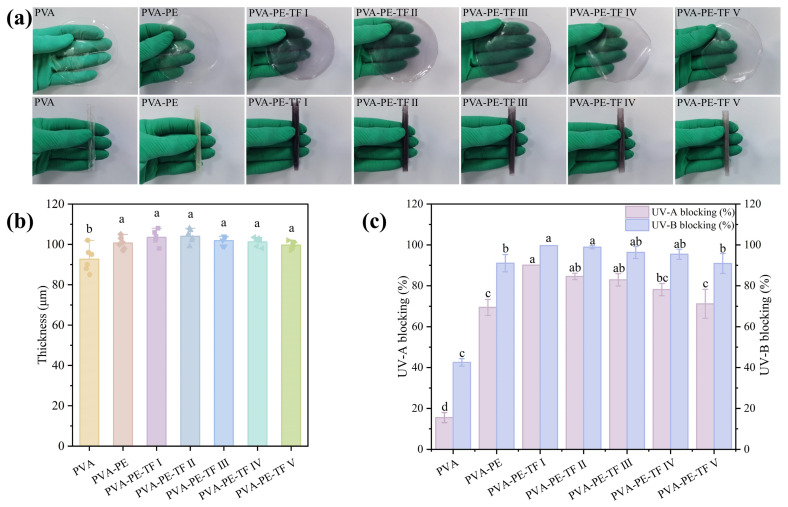
Characterization results of PVA, PVA-PE and PVA-PE-TF films. (**a**) Appearance; (**b**) Thickness; (**c**) UV-blocking. Data are shown as mean ± standard deviation (*n* = 3), where *n* represents the number of independent replicates for each measurement. Different superscript letters in the figures indicate statistically significant differences (*p* < 0.05).

Figure 4a displays the appearance and flexibility of the films. Film thickness is a critical parameter influencing the structural integrity and barrier performance of packaging materials. Figure 4b shows the variation in the average thickness among the different films. The results demonstrate that the thickness of the composite films containing TA and Fe^3+^ ranged from 99.67–101.83 μm, whereas PVA and PVA-PE films exhibited thicknesses of 92.67 μm and 100.67 μm, respectively, indicating the PVA-PE-TF films were thicker than PVA and PVA-PE films. This increase in thickness may be attributed to the higher total solids content introduced by the additional components. Color is a key parameter for evaluating the marketability and consumer acceptance of functional films. CIE 1931 colorimetric charts were used to visually represent the color coordinates of films. As shown in Figure S2a, PVA and PVA-PE films appeared mainly in the white and yellow–white regions, whereas films containing TA and Fe^3+^ shifted toward purplish-red, with PVA-PE-TF I -V films exhibiting progressively deeper coloration as TA and Fe^3+^ contents increased. This shift may result from the metal coordination between the polyphenolic hydroxyl groups of TA and Fe^3+^, forming a stable purple-red complex [35].

The UV-shielding performance of packaging films is important for suppressing photooxidative discoloration, nutrient degradation, and lipid oxidation in foods exposed to high-energy radiation. Although the ozone layer filters out nearly all UVC radiation (200–280 nm), UVA (320–400 nm) and UVB (280–320 nm) can still induce oxidative reactions in food components and generate undesirable degradation products [36]. To evaluate the optical shielding properties of the prepared films, their transmittance was measured over the wavelength range of 200–800 nm. As shown in Figure S2b and Figure 4c, the PVA film exhibited high UV transmittance, with UVA and UVB blocking efficiencies of only 15.5% and 42.5%, respectively. In contrast, the UVA and UVB blocking efficiencies of PVA-PE-TF I reached 90.1% and 99.6%, respectively. PVA-PE-TF II–V films also exhibited strong UV-shielding performance, with UVB transmittance below 9.5% and UVA transmittance below 29.6%.

The enhanced UV-blocking performance may be attributed to multiple synergistic mechanisms. First, the aromatic phenolic constituents in the EO and the galloyl-rich conjugated structures of TA can act as UV-absorbing chromophores. Their delocalized π-electron systems undergo electronic excitation after absorbing UV photons, thereby reducing the UV energy transmitted through the films [37,38]. Coordination between TA and Fe^3+^ further alters the electronic structure of the galloyl groups and induces broad ligand-to-metal charge-transfer absorption, thereby enhancing light attenuation in both the UVA and UVB regions [11]. Second, the CNC-stabilized emulsion droplets and CNC-rich microdomains form numerous heterogeneous interfaces with refractive-index differences within the PVA matrix. Multiple light scattering at these interfaces increases the optical path length within the films, thereby enhancing the probability of UV absorption [39]. Therefore, the UV-shielding effect of the composite films does not originate from a single component, but rather from the combined contributions of molecular absorption and interfacial scattering. In addition, hydrogen bonding among PVA, CNC, and TA, together with TA–Fe^3+^ coordination, forms a stable dual-crosslinked network. This network restricts polymer-chain mobility and helps maintain the uniform distribution of UV-active components within the films. Consequently, after 30 days of irradiation, the UV transmittance of the PVA-PE and PVA-PE-TF films remained below 50%, as shown in Figure S2c. The network may also retard UV-induced polymer-chain scission and structural relaxation, thereby maintaining the long-term UV-shielding performance of the films. In contrast, the PVA film almost completely lost its UV-shielding capability after 30 days of irradiation, which may be associated with photodegradation of its hydroxyl-rich polymer backbone.

#### 3.2.2. Chemical Structure Analysis

**Figure 5:**
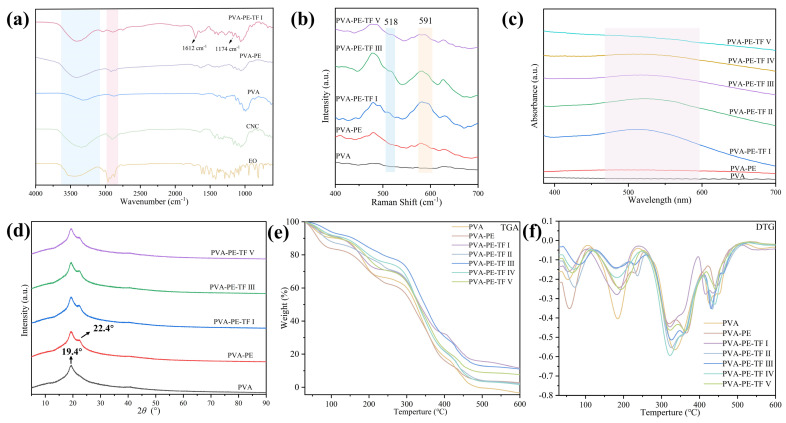
Characterization results of PVA, PVA-PE and PVA-PE-TF films. (**a**) FT-IR spectra; (**b**) Raman spectra; (**c**) UV-vis spectra; (**d**) XRD; (**e**) TGA curves; (**f**) DTG curves.

FT–IR is an effective technique for identifying functional groups and analyzing intermolecular interactions. The absorption spectra of PVA, PVA-PE, and PVA-PE-TF I films are presented in Figure 5a. For EO, the broad absorption band at approximately 3200–3500 cm^−1^ indicates the overlapping stretching vibrations of –OH groups. The peaks observed between 2875 and 2960 cm^−1^ are attributed to the C–H stretching vibrations of –CH_3_ and –CH_2_ groups. The peaks at 1620, 1584, 1517, and 1458 cm^−1^ correspond to the skeletal vibrations of aromatic benzene rings [1]. For PVA film, the absorption band near 3300 cm^−1^ corresponds to the stretching vibration of –OH groups. The signal at 2891 cm^−1^ originates from C–H_2_ stretching vibrations, while the peaks at 1429 and 1029 cm^−1^ are assigned to C–H bending and C–OH stretching vibrations, respectively [40]. However, in the PVA–PE film spectrum, several characteristic peaks of EO disappear or weaken. In particular, the peak at 1029 cm^−1^ associated with C–OH in PVA-PE is significantly weakened, indicating that EO is successfully encapsulated within the composite film matrix. In addition, compared with PVA, the –OH absorption band of PVA-PE exhibits a blue shift from 3314 cm^−1^ to approximately 3430 cm^−1^. This shift suggests the change in intermolecular interactions, such as hydrogen bonding, between the phenolic hydroxyl groups of EO and PVA [41]. Notably, the spectra of PVA-PE and PVA-PE-TF I are highly similar, that the principal chemical framework of the PVA-based matrix was retained after the addition of TA and Fe^3+^. In addition, bands near 1612 and 1174 cm^−1^ were observed in PVA-PE-TF I and were assigned to the aromatic C=C skeletal vibration and phenolic C–O stretching vibration of TA, respectively [42], supporting the incorporation of TA into the film matrix. Changes in the position, broadening, and profile of the O–H stretching region further suggested that TA and Fe^3+^ altered the intermolecular inter-action environment through competitive hydrogen bonding and coordination interactions.

To verify the coordination interaction between Fe^3+^ and TA, Raman spectroscopy was employed to analyze the Fe^3+^-TA complexes (Figure 5b). Compared with the PVA and PVA-PE films, the PVA-PE-TF films exhibited new peak at approximately 591 cm^−1^ and near 518 cm^−1^, which are attributable to MPN between TA and Fe^3+^ [19]. In addition, as shown in the UV–vis spectra (Figure 5c), a new absorption band appeared at around 535 nm for the PVA-PE-TF films, whereas this feature was absent in the PVA and PVA-PE films. It is well established that Fe^3+^-catechol complexes display characteristic absorption maxima at approximately 759, 575, and 492 nm for mono-, bis-, and tris-coordinated species, respectively [12]. Therefore, the observed absorption in the range of 530–570 nm indicates the coexistence of bis- and tris-coordinated MPN complexes in the system. The formation of this MPN can enhance the mechanical properties of the film. Figure S2d presents the XPS spectra of the PVA, PVA-PE, and PVA-PE-TF film samples. Characteristic C1s (near 286 eV) and O1s (near 533 eV) peaks were observed in all films. The O1s spectra were baseline-corrected to clarify oxygen species, with C–O at 533 eV and C=O at 531.5–532 eV identified. As shown in Figure S2e–i, this deconvolution reveals two characteristic peaks assigned to C–O and C=O species, confirming their presence in all film samples. In the case of the PVA film, the binding energies were approximately 531.85 eV for C–O and 533 eV for C=O, with corresponding peak area ratios of 79.52% and 20.48%, respectively, indicating the abundance of oxygenated groups in PVA [43]. After the introduction of the emulsion, the binding energy of the C–O bond shifted to 531.35 eV, and its peak area ratio increased to 93.17%. This change was mainly attributed to the incorporation of hydroxyl-rich CNC into the PVA film matrix [44]. For the PVA-PE-TF I, III, and V films, the binding energies of the C–O bond were 531.2 eV, 531.4 eV, and 531.9 eV, respectively, while the corresponding peak area ratios increased to 97.40%, 95.22%, and 95.42%. Conversely, the proportion of the C=O component decreased to below 5%. These variations were ascribed to the chelation between TA and Fe^3+^, leading to the formation of Fe–O bonds and indicating the successful construction of the MPN [45]. This result is consistent with the FT-IR, Raman and UV-vis analysis results.

#### 3.2.3. XRD Analysis

Understanding the crystalline characteristics of film materials is essential, as these features fundamentally influence their mechanical strength and barrier performance. Therefore, X-ray diffraction was employed to analyze the films. Figure 5d presents the XRD patterns of the films, which display distinct structural features. The PVA film exhibited a broad diffraction peak at 2θ = 19.4°, corresponding to the (101) plane, indicating its typical semicrystalline nature. This diffraction signal arises from the amorphous regions of PVA. For the PVA-PE system, a new diffraction peak emerged at 2θ = 22.4°, assigned to the (200) plane, whereas the original peak at 19.4° remained unchanged, indicating that the crystalline structure of the CNC was preserved and that the PVA and CNC were highly compatible [7]. In the PVA-PE-TF I, PVA-PE-TF III, and PVA-PE-TF V curves, the diffraction peaks of PVA and CNC remained detectable, likely due to hydrogen-bond interactions between PVA and CNC that did not disrupt their crystal structures.

#### 3.2.4. TGA Analysis

In this study, the thermal stability of the films was characterized by TGA through recording the mass changes in the samples during continuous heating. The TGA and derivative DTG curves (Figure 5e,f) reflected the thermal degradation behavior of the PVA, PVA-PE, and PVA-PE-TF series films over the temperature range of 30–600 °C. The thermal stability of the films was mainly affected by factors such as fiber size, chemical composition, crystal structure, and hydrogen-bonding interactions. In addition, the dispersion uniformity of the fillers within the polymer matrix and the interfacial bonding strength between the fillers and the matrix also significantly affected the thermal resistance of the films [46].

During the initial stage (30–130 °C), the mass loss of the films was mainly attributed to the evaporation of absorbed water and a small amount of EO [37]. The second stage occurred at 130–270 °C and may have been associated with the decomposition of the EO and glycerol. The third stage was the main thermal degradation stage of the films, mainly involving the decomposition of PVA macromolecular chains and the gradual destruction of the crosslinked network. As shown in Figure 5e, the PVA film exhibited the greatest overall extent of thermal degradation. Among all the PVA-PE-TF composite films, PVA-PE-TF I had the highest residual mass at 600 °C, followed by PVA-PE-TF III and PVA-PE-TF V, whereas the residual masses of PVA-PE-TF II and PVA-PE-TF IV were relatively close to that of the PVA-PE film. Compared with the PVA film, all PVA-PE-TF I–V films exhibited higher residual masses at 600 °C, indicating that the incorporation of the emulsion, TA, and Fe^3+^ improved the high-temperature char-forming capacity of the films. This may have been associated with hydrogen-bonding interactions among PVA, CNC, and TA, TA–Fe^3+^ coordination, and the reinforcing effect of CNC. These interactions may have restricted the mobility of the polymer chains and promoted the formation of relatively stable carbonaceous residues during heating.

#### 3.2.5. Morphology Analysis

**Figure 6:**
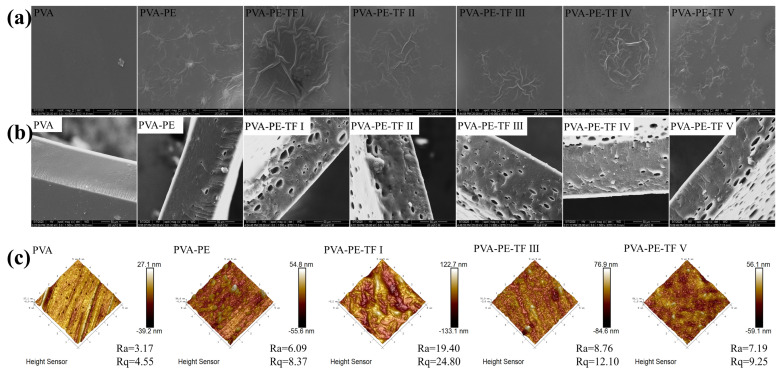
Characterization results of PVA, PVA-PE and PVA-PE-TF films. (**a**,**b**) SEM morphologies of surface and cross-section; (**c**) AFM images.

The SEM of the films are depicted in Figure 6a. The PVA films exhibited a uniform, dense and smooth surface. In comparison, the addition of emulsion resulted in a rougher surface morphology with slight reticulation, which could be attributed to the cross-linking effect between CNC and PVA. Notably, the PVA-PE-TF films exhibited a distinct and compact network structure. Interestingly, as demonstrated in Figure 6b, the cross-sections of films containing emulsion exhibited a sponge-like morphology with apparent micropores. The observed effect likely arises from EO movement within the film matrix, where they tend to migrate, cluster, and volatilize toward the surface during water evaporation. Furthermore, the PVA-PE-TF I film had the most porous structure, probably due to the encapsulation of the most emulsion droplets. These results indicate that the incorporation of a dual cross-linked network stabilizes EO within the film matrix, which further explains the superior barrier properties of the PVA-PE-TF films.

To further investigate the structural and morphological evolution of the films, AFM was employed to examine both surface and cross-sectional features. As displayed in Figure 6c, the PVA film exhibited an average surface roughness (Ra) of 3.17 nm and a root-mean-square roughness (Rq) of 4.55 nm, suggesting a smooth and uniform morphology. The observed invagination structure may originate from the generation of air bubbles during the film molding process. In contrast, the PVA-PE films presented increased surface roughness (Ra = 6.09 nm, Rq = 8.37 nm) and nanorodular structures on the surface, which may be attributed to the hydrogen bonding of CNC-stabilized EO emulsion molecules that prompts the assembly of the emulsion molecules into nanoscale aggregates, as well as the agglomeration phenomenon of nanoparticles during the drying process. Furthermore, the surface roughness values of the PVA-PE-TF I, II, and III films were measured to be 24.80 nm, 12.10 nm and 9.25 nm, respectively, which were greater than that of the PVA film (4.55 nm). These findings indicate the successful formation of a crosslinked network structure. Additionally, a direct correlation was found between the surface roughness of PVA-PE-TF films and the levels of TA and Fe^3+^, where increased roughness indicated denser hydrogen bonding and enhanced metal–phenol cross-linking. This increase in roughness was attributed primarily to the deposition of CNC-TA-Fe^3+^ nanocomposites on the film surface [43]. These results are consistent with those from the SEM analysis. The composite films’ increased surface roughness facilitate stress transfer [47].

#### 3.2.6. WCA, WVP and OP Analysis

**Figure 7:**
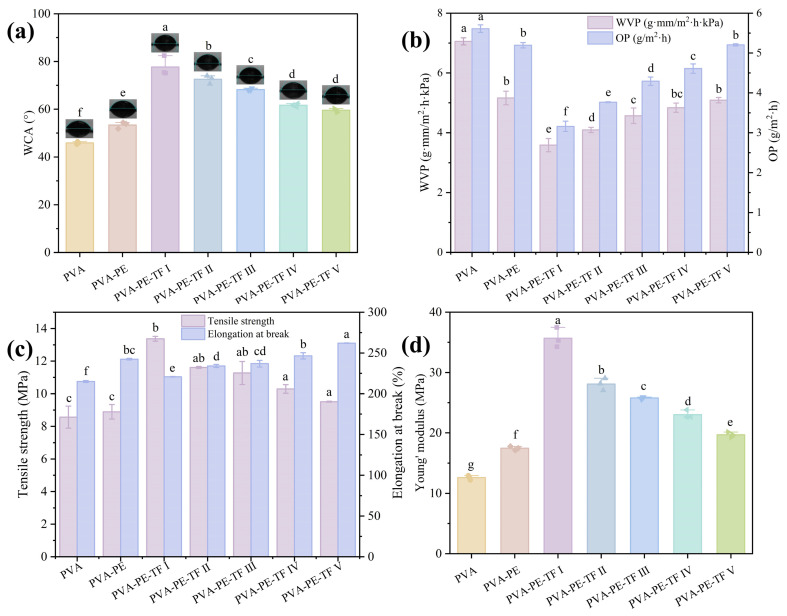
Characterization results of PVA, PVA-PE and PVA-PE-TF films. (**a**) WCA; (**b**) Permeability; (**c**) TS and EAB; (**d**) Young’s modulus. Data are shown as mean ± standard deviation (*n* = 3), where *n* represents the number of independent replicates for each measurement. Different superscript letters in the figures indicate statistically significant differences (*p* < 0.05).

Hydrophobicity serves as a key parameter for evaluating the effectiveness of preservation packaging under humid environments, while the WCA is widely applied to characterize surface wettability in terms of hydrophilicity and hydrophobicity. Generally, the WCA lower than 90° indicates hydrophilic materials, while a WCA higher than 90° corresponds to hydrophobic materials. Figure 7a. illustrates that the WCA of the PVA film was the lowest (45.7°), reflecting its strong hydrophilicity. This property is likely attributed to abundant hydroxyl groups within its structure, which readily interact with water molecules through hydrogen bonding, thereby enhancing surface wettability [48]. With the addition of emulsion, the WCA of the PVA-PE film slightly increased (53.4°), which may be attributed to the embedding of EO in emulsion, which is an oily mixture with strong hydrophobicity, thus increasing the hydrophobicity of the film surface. However, the hydrophilicity of the PVA-PE film was still, probably because of the exposed hydroxyl groups in the CNC molecular structure as well as the presence of polar sulfate groups, which can easily interact with water molecules and increase the overall hydrophilicity of the film [49]. Notably, the WCA of the composite films increased with increasing of TA and Fe^3+^ contents, from 59.5° to 77.7°. The PVA-PE-TF I film had the highest contact angle (77.7°). Compared to the PVA and PVA-PE groups, the composite films exhibited higher overall hydrophobicity on their surfaces. This may be related to the fact that the TA and Fe^3+^ complexes filled the microscopic pores of the membrane layer, which reduced the space available for the adsorption of free water, thus lowering the water content of the film. Moreover, in addition to forming a stable MPN with Fe^3+^, the polyphenolic hydroxyl groups in TA can also establish hydrogen bonds and interfacial interactions with the hydroxyl groups of CNC molecules. These interactions stabilize the film by generating a dual-crosslinked network. This structure can effectively shield the hydrophilic groups on the CNC surface, limit their contact with water molecules, increase the hydrophobicity, and consequently increase the WCA [50]. TA-Fe^3+^ complexes can construct a dual-crosslinked network structure crosslinked by hydrogen bonding and MPN in the film and regulate the surface wettability of the composite films. This structural modulation behavior has potential application and popularization value in food packaging, functional coatings, and other fields with specific requirements for hydrophobicity.

Establishing an effective water vapor barrier is crucial for minimizing moisture exchange between food products and their environment, thereby contributing to flavor preservation, enhanced product stability, and prolonged shelf life. For dry and hygroscopic goods, a low WVP is preferred, which indicates stronger resistance to ambient moisture transmission, whereas for high-moisture products, moderate moisture transmission is beneficial for minimizing vapor condensation inside the packaging system [15]. In Figure 7b, the WVP of the PVA film was 7.06 g·mm/m^2^·h·kPa. After adding the emulsion, the WVP of the PVA-PE film decreased to 5.16 g·mm/m^2^·h·kPa. The reduced WVP is attributed primarily to the presence of emulsions, which increased the tortuosity of the internal film network and thereby prolonged the migration route of water molecules. In addition, the spatial distribution of oil droplets further hinders molecular movement, effectively impeding their transmembrane transport. Notably, among all the samples, the PVA-PE-TF I film presented the lowest WVP value (3.59 g·mm/m^2^·h·kPa). One factor contributing to the reduced WVP of the PVA-PE-TF films is the hydrogen bonding formed by the phenolic hydroxyls in TA with hydroxyl functionalities on CNC. It is generally believed that this interaction reduces the free volume within the matrix, resulting in a denser network structure and improved resistance to WVP, which serves a pivotal function in mitigating both chemical degradation and microbial spoilage in packaging materials for foods [22]. Additionally, the incorporation of MPN improved the affinity of the material for hydrophilic groups, facilitating the formation of a hydration layer consisting of a small number of water molecules that inhibits water vapor transmission. Films with low OPs can effectively inhibit oxygen-induced food reactions, thus enhancing the shelf stability of food products. As shown in Figure 7b, the PVA film has the highest OP (5.61 g/m^2^∙h) and the PVA-PE film has an OP of 5.20 g/m^2^∙h. This may be attributed to the homogeneous dispersion of the CNC particles in the PVA-PE film, which act as “nanobricks” structures in the film, thus reducing the voids within the film matrix [51]. The PVA-PE-TF film had a reduced OP (3.16–5.20 g/m^2^∙h), in particular, the PVA-PE-TF I film had the lowest OP value (3.16 g/m^2^∙h). This may be due to the synergistic cross-linking of CNC, TA, and Fe^3+^ through hydrogen bonding and metal complexation forms a dual-crosslinked network, enhancing the polymer chain alignment of the composite film and consequently improving its barrier properties.

#### 3.2.7. Mechanical Properties Analysis

Packaging films with increased mechanical strength are crucial for resisting external forces during transportation and storage, thereby maintaining the physical integrity of the food product. For the evaluation of mechanical performance, TS, stress–strain profile, EAB, and Young’s modulus were determined. The TS of PVA-PE-TF I-V reached 13.37 MPa, 11.61 MPa, 11.27 MPa, 10.29 MPa and 9.51 MPa, which were 56.2%, 35.6%, 31.7%, 20.2% and 11.1% greater than those of PVA (8.56 Mpa), respectively (Figure 7c). The Young’s modulus of the PVA-PE-TF V-I film increased from 19.67 MPa to 35.69 MPa (Figure 7d), indicating that the incorporation of emulsion, TA, and Fe^3+^ substantially enhanced the mechanical properties of the film. This improvement is attributed to the TA can form MPNs with Fe^3+^ via three gallic acid groups. TA can also form noncovalent interactions with the hydroxyl groups of CNC via its phenolic hydroxyl groups [52]. Hydrogen bonding and MPNs are formed within the PVA matrix, constructing a dense dual cross-linked network structure. This structure achieves efficient intermolecular stress transfer and strengthens polymer interchain interactions, thereby enhancing the structural stability and mechanical properties of composite films. Moreover, the electrostatic interactions between the Fe^3+^ and the OSO_3_^−^ in CNC also contribute to the TS to a certain extent [53]. Nevertheless, the stress–strain curves and EAB date of the films revealed that the corresponding EAB values range from 261.99% to 220.80%, with PVA-PE at 242.35%, all of which are higher than that of PVA (215.04%) (Figure S2j). This is attributed to the introduction of hydroxyl-rich CNC and TA, which provide a large number of hydrogen-bond active sites, thereby dissipating the externally applied mechanical energy [50]. However, the EAB and strain values of the PVA-PE-TF films are inversely proportional to the concentrations of added TA and Fe^3+^. This is likely because the formed MPNs further restricts the freedom of movement of the polymer chains, thereby reducing the flexibility of the composite film. This further confirms the role of MPN in regulating the stiffness and flexibility of the composite film.

### 3.3. pH-Responsive Release Behavior Analysis

**Figure 8:**
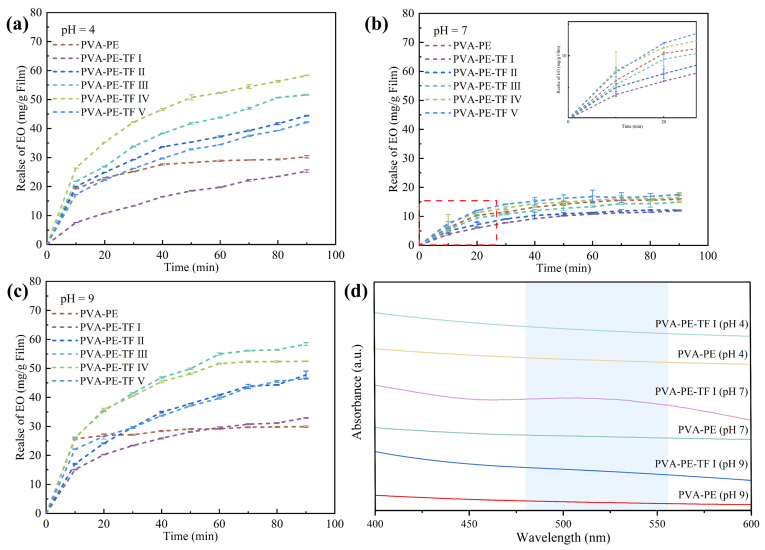
Characterization results of PVA, PVA-PE and PVA-PE-TF films. (**a**–**c**) pH-responsive release; (**d**) pH-dependent UV-vis spectra of MPN. Data are shown as mean ± standard deviation (*n* = 3), where *n* represents the number of independent replicates for each measurement.

Changes in pH are important indicators of food spoilage. Most fruits and vegetables undergo acidification, whereas meat products produce alkaline ammonia, increasing the pH. As shown in Figure 8a–c, compared with the conditions at pH = 7, the films rapidly release large amounts of EO at pH = 4 and pH = 9 to inhibit microorganisms and preserve food. Specifically, among all the PVA-PE-TF (I-V) films, the EO release levels reached 32.98–58.4 mg/g at pH = 4, 11.77–17.74 mg/g at pH = 7, and 33.11–58.4 mg/g at pH = 9. Compared with neutral conditions, EO release increased by 179.9–229.2% under acidic conditions and by 181.3–229.2% under alkaline conditions. Figure 8d further demonstrates the pronounced pH dependence of the MPN. Under acidic and alkaline conditions, the MPN structure was severely disrupted, whereas under neutral conditions, the characteristic absorption band of MPN in the range of 450–600 nm was retained. This may be because under acidic conditions, the emulsion in the film breaks down and the large amount of H+ in the environment causes protonation of the hydroxyl groups, altering the surface charge distribution and reactivity of the polymer molecules, which would cause the MPN to collapse, thereby releasing the EO encapsulated within the films [25]. Furthermore, the coordination number of the TA-Fe^3+^ complex is pH-dependent. Under acidic conditions, mono-complexes or, preferably, bis-complexes are formed, whereas at neutral pH, more stable bidentate or tridentate complexes readily form. Moreover, under alkaline conditions, the high concentration of hydroxide ions may interfere with MPN by competitively interacting with metal centers, thereby weakening or partially disrupting the integrity of the MPN. Simultaneously, deprotonation of OH in TA may enhance electrostatic repulsion between phenolate anions, disturb the original hydrogen-bond balance, and consequently lead to network loosening and pore enlargement [12]. These scenarios may lead to the massive release of EO from the film. Figure 8a shows that the amount of EO released from the PVA-PE-TF I-IV films was inversely proportional to the concentrations of TA and Fe^3+^ added. This may be because the lower the concentration is, the weaker the interaction between Fe^3+^ and TA molecules, resulting in a less stable dual-crosslinked network structure, which makes the release of EO molecules easier. However, the PVA-PE-TF V film exhibited a weak EO release capability, possibly due to the larger pore size of the formed network structure, which resulted in a reduced EO delivery capacity. Additionally, the EO in the PVA-PE film was almost completely released by approximately 40 min. As shown in Figure 8b, increasing the concentration of the PVA-PE-TF film slows down the release rate of EO, suggesting that the incorporation of TA and Fe^3+^ enhanced the retention of EO within the matrix, thereby extending the service life of the film. As shown in Figure 8c, at pH = 9, the PVA-PE film rapidly released completely within 10 min. This may be attributed to the weak molecular interactions in the PVA-PE film, resulting in a weaker ability to control the release of EO. Compared with traditional film materials, this pH-responsive characteristic reduces EO loss, avoids the adverse effects of excessive EO on food sensory quality, and achieves sustained freshness preservation. These results demonstrate that the prepared film has pH-responsive release properties, which are highly beneficial for achieving the controlled release of EO to extend the shelf life of physical products. The Peppas model was employed to determine the release exponent (*n*) of different films (Tables S1–S3). The PVA-PE films exhibited Fickian diffusion behavior (*n* < 0.45), indicating that EO release is primarily governed by concentration-driven diffusion. In contrast, the PVA-PE-TF I films showed *n* values characteristic of anomalous (non-Fickian) transport (0.89 > *n* > 0.45) under pH = 4 and pH = 7 conditions, suggesting that EO release is controlled by a coupled mechanism involving both diffusion through the polymer matrix and relaxation/swelling of the dual cross-linked network [54]. However, at pH = 9, the *n* value of the PVA-PE-TF I films decreased below 0.45, indicating a transition to Fickian diffusion due to partial disruption of the dual cross-linked network structure. This behavior confirms that the interpenetrating CNC-mediated hydrogen-bonded network and the TA-Fe^3+^ coordination network provide stronger confinement on EO migration under acidic and neutral conditions. The pH-responsive properties expand its potential applications in the field of smart food preservation.

### 3.4. Antioxidant and Antimicrobial Activities Analysis

**Figure 9:**
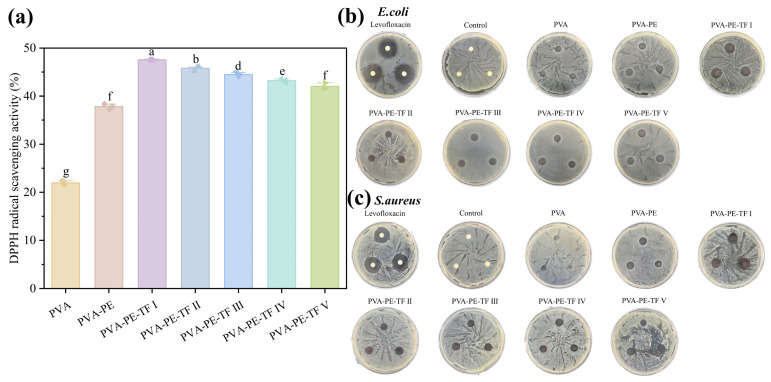
Antioxidant and antimicrobial activities of PVA, PVA-PE and PVA-PE-TF films. (**a**) Antioxidant activity. (**b**) Antimicrobial activity against *E. coli*; (**c**) Antimicrobial activity against *S. aureus* (the inhibition zone in the upper left corner of each plate is a positive control). Data are shown as mean ± standard deviation (*n* = 3), where *n* represents the number of independent replicates for each measurement. Different superscript letters in the figures indicate statistically significant differences (*p* < 0.05).

Lipids contribute to the flavor of food products; however, the double bonds in unsaturated fatty acids make them highly reactive and prone to oxidation when exposed to heat, oxygen, or humidity. This oxidation accelerates quality deterioration, causes flavor loss, and may compromise food safety. The antioxidant potential of the films was evaluated using the DPPH radical-scavenging technique, and the quantitative results are presented in Figure 9a. The PVA film presented a relatively low radical-scavenging rate of 21.8%, indicating limited intrinsic antioxidant function. Following the addition of EO-containing emulsion, the film exhibited a radical-scavenging activity of 37.2%. This increase may result from bioactive phenolic constituents in the EO, primarily carvacrol, which possess hydroxyl groups capable of donating hydrogen atoms. These groups facilitate the stabilization of free radicals via electron delocalization, thereby disrupting oxidative chain propagation. Moreover, the electrophilic characteristics of these phenolics contribute to the neutralization of ROS, further reinforcing the antioxidant properties of EO in composite film matrices [37]. The system exhibited increased free radical scavenging after integration of TA and Fe^3+^. The scavenging rates of the resulting composite films (I-V) reached 47.4%, 45.4%, 44.4%, 43.2%, and 41.7%, respectively. This enhancement may be attributed to the abundant phenolic hydroxyl groups in TA, which effectively quench DPPH free radicals through an electron-proton transfer mechanism [55]. It may also result from the MPNs that stabilize phenoxy radicals and regulate redox behavior, thereby extending antioxidant activity. These results indicate that the improvement in the antioxidant properties of the composite films does not stem from a single active component, but rather from the synergistic effect between the phenolic substances of the EO and the MPN.

Microbial contamination is a major contributor to the degradation and spoilage of food products. A promising strategy for addressing this issue involves the creation of bioactive packaging with antimicrobial functionality, which can prolong the shelf lives of perishable items. EO exhibits broad-spectrum antibacterial efficacy. Accordingly, the antimicrobial activity of the EO renders it a promising candidate for applications in food preservation, pharmaceutical formulations, and natural antimicrobial delivery systems [15]. A disk diffusion test was performed to determine the antimicrobial properties of the fabricated films using *E. coli* and *S. aureus* as model strains. As illustrated in Figure 9b,c and Table 2, the PVA films exhibited minimal inhibition zones against both bacteria (8.97 mm and 8.08 mm, respectively), indicating limited inherent antimicrobial activity. Upon incorporation of the emulsion loaded with EO, the inhibition zones increased to 10.99 mm against *E. coli* and 11.22 mm against *S. aureus*. These increases are attributed to the fact that carvacrol, the primary component of the EO, disrupts bacterial membranes, thereby compromising cell wall integrity and promoting the leakage of intracellular components [56]. Moreover, the needle-like shape of CNC (Figure S3) may exert mechanical pressure when interacting with bacterial cell membranes, which could result in structural disruption or deformation of the cells [57]. Notably, the incorporation of TA and Fe^3+^ further enhanced the antimicrobial activity of the composite films. The inhibition zones against *E. coli* increased to 10.58–14.88 mm, while those against *S. aureus* expanded to 9.94–15.76 mm, with inhibition diameters positively correlated with the concentrations of TA and Fe^3+^. The PVA-PE-TF I film exhibited the strongest antibacterial performance, with inhibition zones against *E. coli* and *S. aureus* enlarged by 5.91 mm and 7.68 mm, respectively, compared with the PVA. The enhancement of antibacterial efficacy is likely attributed to the synergistic antimicrobial effect of the TA and EO.

### 3.5. Application of Composite Films in Yam Preservation Analysis

#### 3.5.1. Preservation Performance of Yam Analysis

**Figure 10:**
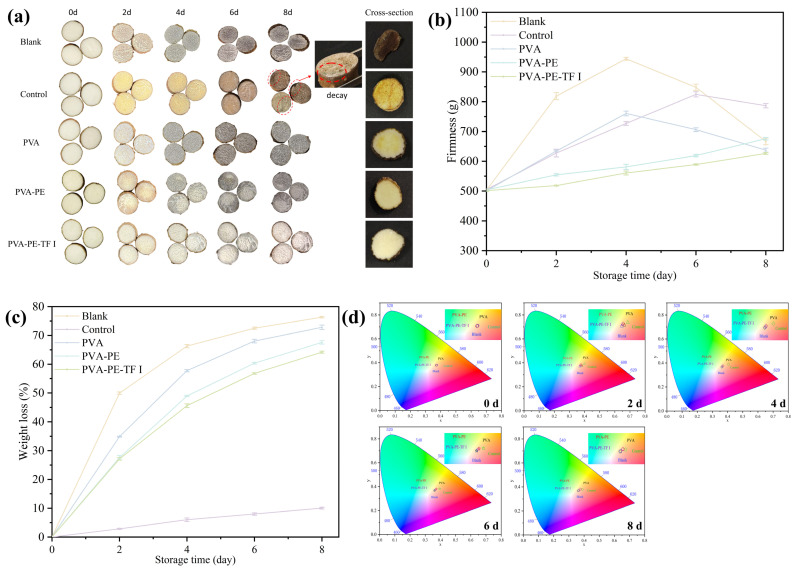
Preservation performance analysis of yam treated with Blank, Control, PVA, PVA-PE and PVA-PE-TF films during storage. (**a**) Appearance; (**b**) Firmness; (**c**) Weight loss; (**d**) CIE (Blank: no film treatment; Control: plastic wrap treatment). Data are shown as mean ± standard deviation (*n* = 3), where *n* represents the number of independent replicates for each measurement. Different superscript letters in the figures indicate statistically significant differences (*p* < 0.05).

The effects of PVA-PE and PVA-PE-TF composite films with excellent mechanical strength, barrier properties, and antioxidant and antimicrobial activities on the preservation of yam were systematically investigated. Quality parameters including appearance, color, and browning index, firmness, weight loss were monitored during storage. As shown in Figure 10a, yams in the Blank, Control, PVA, and PVA-PE groups exhibited visible browning as early as Day 2, and the degree of browning intensified markedly with prolonged storage. By Day 8, evident decay and spoilage were observed in the Control group. In contrast, yams packaged with the PVA-PE-TF I film maintained a relatively intact appearance, exhibiting a substantially delayed browning process and no spoilage. Changes in firmness further reflected the preservation efficacy of the different films (Figure 10b). During the early storage stage (Days 0–4), all samples exhibited an increase in firmness, which was mainly associated with the natural loss of moisture in the yam and the resulting hardening of its tissue. This trend was more pronounced in the Blank and PVA groups due to more severe dehydration. From Day 4 onward, the firmness of yams in the Blank, Control, and PVA groups declined significantly, which was likely attributable to microbial proliferation and degradation of cell wall polysaccharides, ultimately leading to tissue softening and quality deterioration [36]. In contrast, yams packaged with the PVA-PE and PVA-PE-TF I film maintained relatively stable firmness throughout the storage period. This stability may be attributed to the reduced WVP of the composite films, which limited excessive moisture migration and thereby helped preserve cell wall integrity. Moreover, the incorporation of antimicrobial EO and TA likely contributed to delayed textural degradation of yam during storage by inhibiting microbial growth.

**Figure 11:**
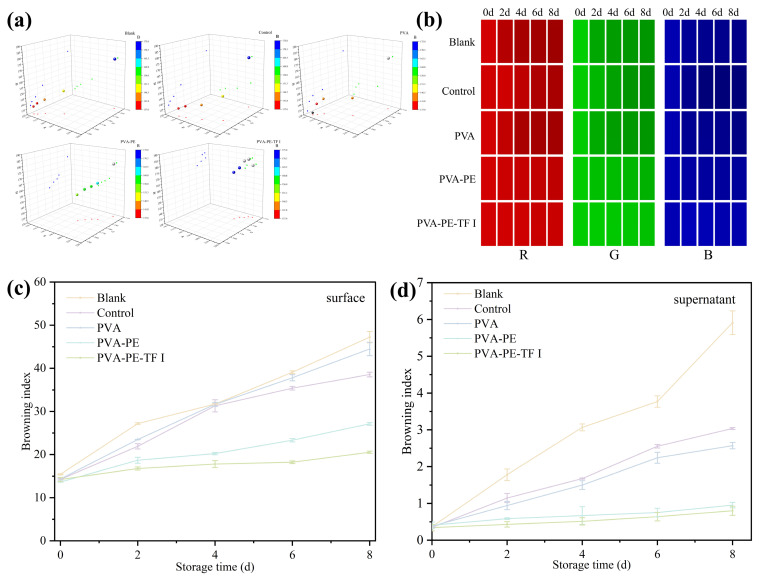
Preservation performance analysis of yam treated with Blank, Control, PVA, PVA-PE and PVA-PE-TF films during storage. (**a**) Three-dimensional RGB; (**b**) Two-dimensional RGB; (**c**) BI of yams’ surface; (**d**) BI of yams’ supernatant. Data are shown as mean ± standard deviation (*n* = 3), where *n* represents the number of independent replicates for each measurement. Different superscript letters in the figures indicate statistically significant differences (*p* < 0.05).

**Figure 12:**
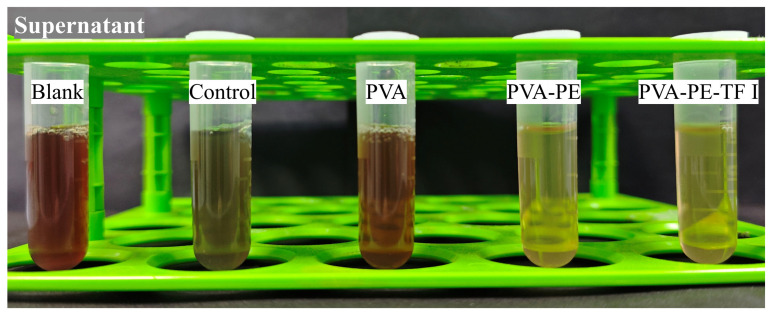
Preservation performance analysis of yam treated with Blank, Control, PVA, PVA-PE and PVA-PE-TF films during storage. Supernatant of yams after 8 days of storage.

As shown in Figure 10c, all samples exhibited a continuous increase in weight loss during storage. The Blank group showed the most severe weight loss, reaching 76.33% by Day 8, indicating extensive dehydration and loss of commercial value. The PVA group also showed a high weight loss rate (72.82%), suggesting that PVA films provided limited preservation benefits under room temperature conditions. Although the Control group maintained relatively stable weight throughout storage, visible spoilage occurred by Day 8, which may be attributed to excessive barrier properties that restricted moisture exchange and led to internal condensation, thereby creating favorable conditions for microbial growth. In contrast, yams packaged with the PVA-PE-TF I film exhibited a reduced weight loss of 64%. This improvement is likely associated with the balanced permeability of the PVA-PE-TF film, which optimizes water vapor regulation, permits normal respiratory metabolism, and minimizes moisture and nutrient loss. To quantitatively assess surface color stability and browning inhibition, CIE chromaticity analysis, RGB color distribution, and BI measurements were conducted (Figure 10d and Figure 11a,b). During storage, samples in the Blank and Control groups showed pronounced color shifts and dispersion in both CIE and RGB color spaces, indicating severe browning and pigment migration. The PVA and PVA-PE groups exhibited moderate color deterioration. In contrast, yams treated with the PVA-PE-TF I film maintained highly concentrated and stable color distributions throughout the preservation period. Consistently, the Blank group exhibited the highest BI values for both surface tissue and extraction supernatant, confirming extensive enzymatic browning (Figure 11c,d). Both the PVA-PE and PVA-PE-TF I films effectively suppressed browning, with the PVA-PE-TF I film showing the lowest BI values and the most pronounced antibrowning effect, which was in good agreement with the appearance results (Figure 12).

#### 3.5.2. Metabolomic Regulation of Yam Analysis

**Figure 13:**
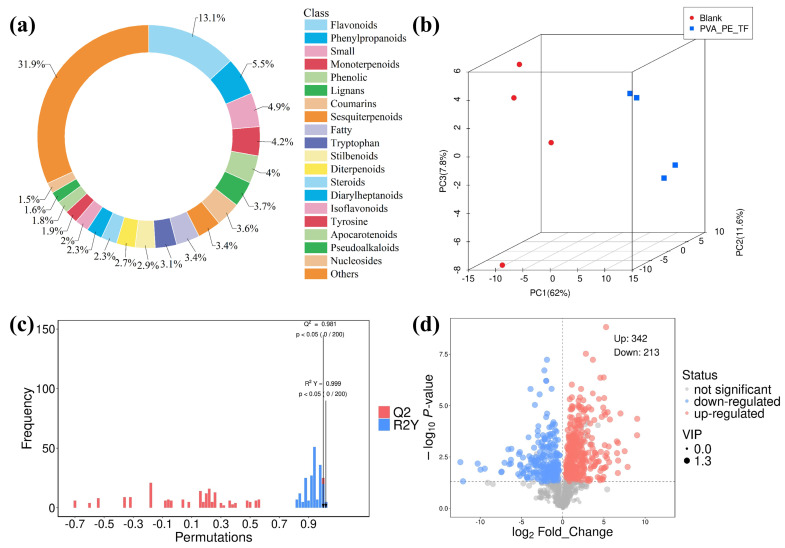
Metabolomic analysis of yam treated with Blank, Control, PVA, PVA-PE and PVA-PE-TF films during storage. (**a**) Categories of differential metabolites; (**b**) PCA; (**c**) OPLS-DA (Q^2^ = 0.981, *p* < 0.05 (0/200); R^2^Y = 0.999, *p* < 0.05 (0/200)); (**d**) Screening of differential metabolites and visualization by volcano plot.

Comprehensive targeted metabolomic analysis was conducted on yams treated with PVA-PE-TF I composite film and untreated control samples. A total of 1021 metabolites were detected, covering flavonoids, phenylpropanoids, oligopeptides, monoterpenes, phenolic acids, lignans, coumarins, sesquiterpenes, fatty acids and derivatives, tryptamine alkaloids, quinolines, diterpenes, steroids, and diarylheptanoids (Figure 13a). Flavonoids and phenylpropanoids were the most abundant, indicating their potential key roles in the physiological processes associated with yam preservation. These metabolite classes are well known for their involvement in plant stress responses and postharvest preservation mechanisms [58]. Metabolic variation across groups and variability within groups were examined using (principal component analysis) PCA [59]. Figure 13b revealed distinct variation patterns in the yam samples preserved under the Blank 1–4 and PVA-PE-TF I 1–4 treatments. Principal components 1 and 2 explained 62% and 11.6% of the total ingredients, respectively, demonstrating that PCA effectively discriminated between the treatment and control groups, with pronounced intergroup differences.

OPLS-DA eliminates metabolite variables that are orthogonal and unrelated to categorical factors, while independently analyzing orthogonal and non-orthogonal components. Notably, the robustness of the OPLS-DA model provides strong statistical support for the biological relevance of the observed metabolic alterations. The exceptionally high predictive ability (Q^2^ = 0.981) and goodness of fit (R^2^Y = 0.999), together with a statistically significant permutation test (*p* < 0.05) (Figure 13c), indicate that the model is highly reliable and free from overfitting. These validation metrics confirm that the clear separation between the control and PVA-PE-TF I treatment groups is driven by systematic and treatment-induced metabolic variation rather than random fluctuations or modeling artifacts.

Volcano plots can visualize the overall distribution of metabolite differences between groups and detect different metabolites. According to [60], metabolites were considered differentially abundant when they met the following thresholds in the OPLS-DA model: VIP > 1, FC ≥ 2 or ≤ 0.5, and *p* < 0.05. Figure 13d shows all the substances detected in this experiment. Each point corresponds to one metabolite, while its size reflects the VIP value in the OPLS-DA model, with larger points indicating higher VIP scores. Red markers denote upregulated metabolites, blue indicate downregulated ones, while gray corresponds to metabolites without changes. According to [61], phenolic compounds and flavonoids are synthesized through the phenylpropane pathway. The presence of these compounds endows plants with the ability to reduce cellular oxidative damage through efficient scavenging of ROS accumulated during abiotic and biotic stress responses. The results revealed that 555 differentially abundant metabolites (342 upregulated and 213 downregulated) were altered in preserved yams, including 65 flavonoids, 33 phenylpropanoids and 33 monoterpenes, among others. Compared with yam samples treated with the blank control, the PVA-PE-TF I composite film likely regulated phenylpropanoid metabolism during yam preservation, thereby activating the yams defense system, and the increase in flavonoid biosynthesis may result from redox reactions within yam metabolic processes, thereby improving its antioxidant capacity and delaying tissue senescence. Furthermore, the film may suppress carbohydrate synthesis and accumulation, effectively reducing enzymatic browning in the yam.

**Figure 14:**
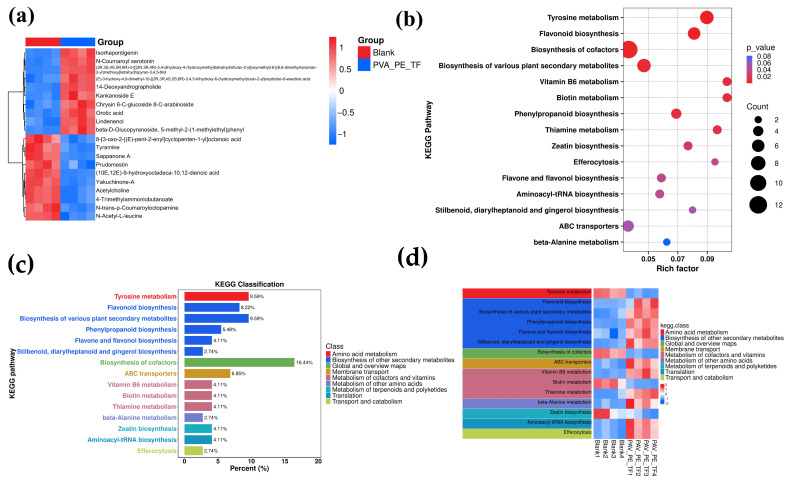
Metabolomic analysis of yam treated with Blank, Control, PVA, PVA-PE and PVA-PE-TF films during storage. (**a**) Heatmap of hierarchical clustering analysis; (**b**) KEGG enrichment bubble; (**c**) KEGG classification; (**d**) KEGG heatmap analysis of differential metabolites (Blank: no film treatment).

Hierarchical cluster analysis was conducted to visualize the differentially abundant metabolites. The top 10 upregulated and downregulated metabolites were selected for clustering. The heatmap applies a color gradient to reflect metabolite abundance, with shades of red corresponding to higher levels and blue to lower levels within each group. As shown in Figure 14a, flavonoids such as isorhapontigenin were markedly upregulated in the treatment group. This increased accumulation of metabolites may be associated with increased antioxidant capacity in yam.

KEGG pathway annotations were applied to classify and visualize the differentially abundant metabolites. Differential metabolites were subjected to KEGG pathway enrichment bubble analysis (Figure 14b). Background colors indicate pathway classifications, while bubble colors denote the relative abundance of differentially abundant metabolites in each pathway, presented on a gradient from high (depicted in deep red) to low (depicted in deep blue). These analyses illustrated the distribution of total differentially abundant metabolite levels across pathways and samples, highlighting metabolic differences between the Blank and PVA-PE-TF I groups. In this study, pathway analysis, based on KEGG pathway annotation of differentially abundant metabolites with enrichment results combined with categorization of pathways revealed that the metabolites were predominantly enriched in multiple metabolic pathways (Figure 14c,d), including amino acid metabolism, secondary metabolite biosynthesis, coenzyme and vitamin metabolism, membrane transport, and energy metabolism. Notably, the pathway of cofactor biosynthesis showed the greatest enrichment ratio (16.44%), suggesting that preservation treatment activated endogenous regulatory mechanisms in yam tissues related to enzyme activity modulation and antioxidant system stabilization. This was followed by enrichments in tyrosine metabolism and biosynthesis of various plant secondary metabolite pathways (both 9.59%), possibly due to a marked response of amino acid metabolism and its derivative secondary metabolic network during yam preservation. These pathways are likely involved in antioxidation, spoilage inhibition, and maintenance of membrane homeostasis [62]. In addition, the pathways for flavonoid biosynthesis and phenylpropanoid biosynthesis were also enriched, indicating that the PVA-PE-TF I composite film induced the accumulation of phenolic compounds in yam. These metabolites not only possess antioxidant and antimicrobial activities and promote cell wall thickening but also may reduce the degree of browning and delay tissue senescence by modulating ROS levels [63]. Moreover, the activation of vitamin B6-related metabolic pathways suggests that during long-term storage, yam tissues actively mobilize vitamin-based antioxidants and coenzyme-related metabolism to maintain cellular homeostasis and metabolic activity [60]. This result reveals the intrinsic metabolic mechanisms underlying the synergistic antisenescence effects achieved via multipathway coordination, providing crucial metabolic insights into the preservation of plant tissues.

## 4. Conclusions

In this study, a pH-responsive dual-network PVA active packaging film was successfully constructed by integrating CNC-stabilized *Mosla chinensis* essential oil (EO) with a tannic acid–Fe^3+^ metal–phenolic network (MPN). Structural characterization by FT-IR, Raman, and UV–vis confirmed the formation of hydrogen-bonding interactions and MPNs within the composite film system. These interactions contributed to improved EO retention, enhanced structural stability of the film matrix, and the construction of a dual-network architecture. The PVA-PE-TF I film achieved UVA and UVB blocking efficiencies of 90.06% and 99.60%, respectively. Its water vapor permeability and oxygen permeability decreased to 3.59 g·mm/m^2^·h·kPa and 3.16 g/m^2^·h, respectively, while the tensile strength increased to 13.37 MPa. In addition, PVA-PE-TF I exhibited enhanced antioxidant activity and broad-spectrum antibacterial performance. The pH-responsive EO release behavior further indicated that the MPN not only reinforced the film structure but also served as a stimulus-responsive dynamic gate for regulating active EO release. Acidic and alkaline environments may induce partial loosening of the MPN, thereby facilitating EO diffusion and release, whereas the relatively stable coordination structure under neutral conditions helped restrict premature EO release. In yam preservation, the enhanced barrier properties, antioxidant and antibacterial activities, and pH-triggered EO release of PVA-PE-TF I collectively mitigated quality deterioration caused by oxidative reactions, moisture migration, and microbial invasion during storage. Metabolomic analysis further suggested that the preservation effect was associated with the regulation of phenylpropanoid metabolism and flavonoid biosynthesis, indicating that the film may not only act through external barrier protection and EO release but also contribute to maintaining antioxidant-related metabolic balance in yam tissues. This work verifies the feasibility of a synergistic strategy integrating CNC-mediated essential oil delivery, MPN-regulated dynamic gating, and active preservation, providing a reference for constructing pH-responsive controlled-release active packaging systems.

## Figures and Tables

**Table 1:** Composition and nomenclature of the composite film samples.

Sample	TA (mg/mL)	FeCl_3_ (mg/mL)	Emulsion (mL)
PVA	–	–	–
PVA-PE	–	–	24
PVA-PE-TF I	10	2.5	24
PVA-PE-TF II	8	2.0	24
PVA-PE-TF III	6	1.5	24
PVA-PE-TF IV	4	1.0	24
PVA-PE-TF V	2	0.5	24

**Table 2:** Inhibition zones of emulsions against *E. coli* and *S. aureus*.

Samples	*E. coli* (mm)	*S. aureus* (mm)
Control	6.00	6.00
Levofloxacin	27.83 ± 1.96 ^a^	22.40 ± 0.42 ^a^
PVA	8.97 ± 0.35 ^d^	8.08 ± 0.32 ^e^
PVA-PE	10.99 ± 0.26 ^c^	11.22 ± 0.71 ^c^
PVA-PE-TF I	14.88 ± 0.69 ^b^	15.76 ± 1.31 ^b^
PVA-PE-TF II	11.37 ± 0.59 ^c^	11.51 ± 0.72 ^c^
PVA-PE-TF III	11.32 ± 0.25 ^c^	11.02 ± 0.34 ^c^
PVA-PE-TF IV	10.57 ± 0.23 ^c^	10.59 ± 0.40 ^c^
PVA-PE-TF V	10.58 ± 0.38 ^c^	9.94 ± 0.81 ^cd^

Data are shown as mean ± standard deviation (*n* = 3), where *n* represents the number of independent replicates for each measurement. Different superscript letters in the figures indicate statistically significant differences (*p* < 0.05). The 6.00 mm value for the blank control represents the paper disk diameter, with no inhibition beyond the disk edge; therefore, it was excluded from statistical analysis.

## Data Availability

The original contributions presented in the study are included in the article/Supplementary Materials, further inquiries can be directed to the corresponding authors.

## Supplementary Materials

The following supporting information can be downloaded at: https://www.mdpi.com/article/10.3390/foods15132401/s1, Figure S1: Characterization results of CNC. (a) Zeta potential; (b) Particle size; (c) XRD; Figure S2: Characterization results of PVA, PVA-PE and PVA-PE-TF films. (a) CIE; (b) Light transmittance; (c) UV-vis transmission spectra of films after 30 days of UV light exposure; (d) XPS scanning spectra; (e–i) Peak-fitted high-resolution O1 s spectra; (j) Stress–strain curves; Figure S3: TEM characterization of CNC; Table S1: The fitting date of EO release obtained with different mathematical models at pH = 4; Table S2: The fitting date of EO release obtained with different mathematical models at pH = 7; Table S3: The fitting date of EO release obtained with different mathematical models at pH = 9.
