# Supplementary material for: pH-Responsive Dual-Network PVA Films Integrating CNC-Stabilized Mosla chinensis Essential Oil Emulsions for Active Food Packaging
Source: Foods. 2026 Jul 7;15(13):2401. doi: 10.3390/foods15132401 (PMC13362074; doi:10.3390/foods15132401)
Supplement: Supplementary file 1 [file foods-15-02401-s001.zip › foods-4397897-supplementary.pdf]

foods

MDPI

Supplementary Materials

pH-Responsive Dual-Network PVA Films Integrating CNC-Stabilized *Mosla chinensis* Essential Oil Emulsions for Active Food Packaging

Huiqiong Wu, Yuxuan Zhu, Huan Liu, Yingying Deng, Zhipeng Wang, Hongning Liu, Zhe Li * and Liangshan Ming*

Institute for Advanced Study, Key Laboratory of Modern Preparation of TCM, Ministry of Education, Jiangxi University of Chinese Medicine, Jiangxi Nanchang 330004, China; wuhuiqiong1@jxutcm.edu.cn (H.W.); zhuyuxuan@jxutcm.edu.cn (Y.Z.); Liuhuan14@jxutcm.edu.cn (H.L.); dengyingying1@jxutcm.edu.cn (Y.D.); wangzhipeng@jxutcm.edu.cn (Z.W.); 19820002@jxutcm.edu.cn (H.L.)

* Correspondence: 20191004@jxutcm.edu.cn (Z.L.); mingls@jxutcm.edu.cn (L.M.)

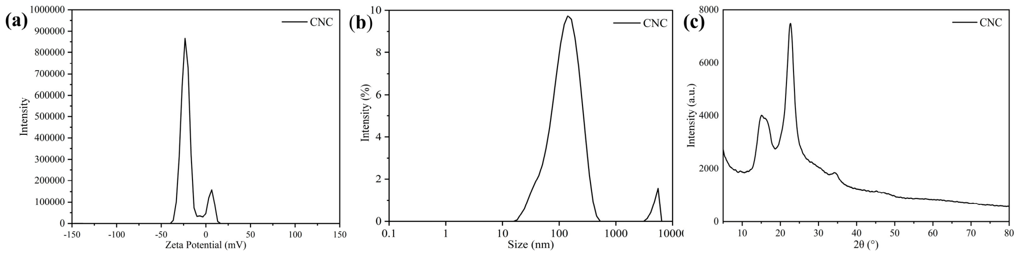

Figure S1. Characterization results of CNC. (a) Zeta potential; (b) Particle size; (c) XRD.

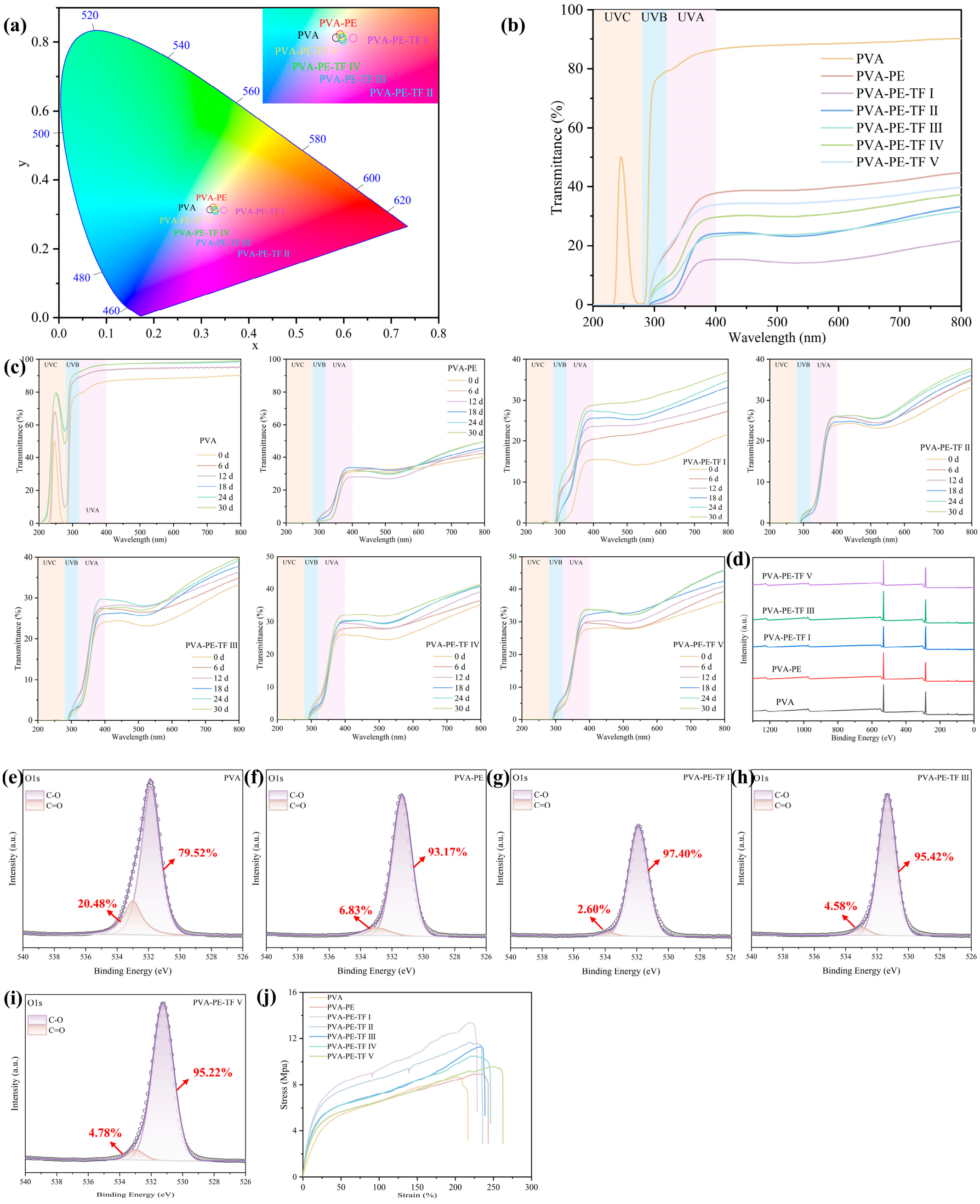

Figure S2. Characterization results of PVA, PVA-PE and PVA-PE-TF films. (a) CIE; (b) Light transmittance; (c) UV-vis transmission spectra of films after 30 days of UV light exposure; (d) XPS scanning spectra; (e-i) Peak-fitted high-resolution O1 s spectra; (j) Stress-strain curves.

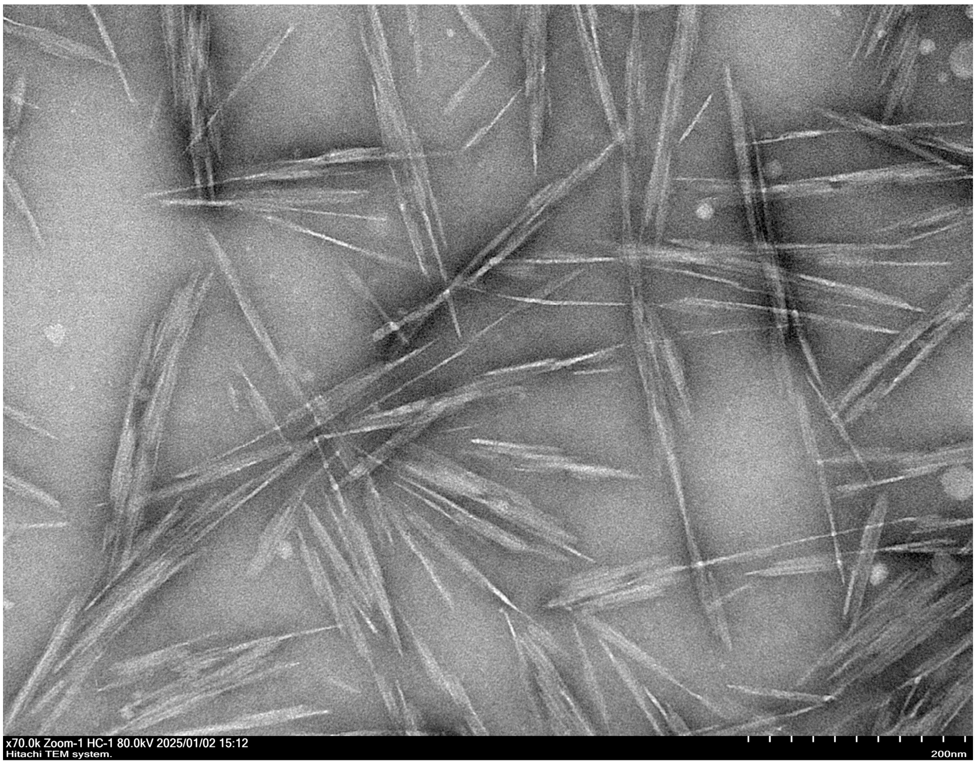

Figure S3. TEM characterization of CNC.

Table S1. The fitting date of EO release obtained with different mathematical models at pH = 4.

Sample	Mathematical Model (pH = 4)			
	**Zero order**	**First order**	**Higuchi**	**Peppas**
PVA-PE	Q = 0.23377t + 13.56294 (R^2 = 0.60097)	Q = 28.87591*(1 - exp(-0.09075*t)) (R^2 = 0.98495)	Q = 2.87758t1/2 + 6.51482 (R^2 = 0.85296)	Q = 12.92174t0.19325 (R^2 = 0.99431)
PVA-PE-TF I	Q = 0.2523t + 4.3801 (R^2 = 0.96879)	Q = 27.86966*(1 - exp(-0.02282*t)) (R^2 = 0.98831)	Q = 2.68651t1/2 - 0.66788 (R^2 = 0.99681)	Q = 2.08195t0.55427 (R^2 = 0.99889)
PVA-PE-TF II	Q = 0.3939t + 12.84408 (R^2 = 0.82554)	Q = 41.80464*(1 - exp(-0.04471*t)) (R^2 = 0.96931)	Q = 4.43164t1/2 + 3.51425 (R^2 = 0.97878)	Q = 8.55671t0.36255 (R^2 = 0.99824)
PVA-PE-TF III	Q = 0.48524t + 13.72085 (R^2 = 0.8584)	Q = 51.17396*(1 - exp(-0.03776*t)) (R^2 = 0.97724)	Q = 5.38356t1/2 + 2.68949 (R^2 = 0.98974)	Q = 8.35273t0.40771 (R^2 = 0.99786)
PVA-PE-TF IV	Q = 0.52365t + 18.68304 (R^2 = 0.78546)	Q = 56.36756*(1 - exp(-0.04964*t)) (R^2 = 0.98804)	Q = 5.9954t1/2 + 5.64488 (R^2 = 0.96445)	Q = 13.0206t0.33783 (R^2 = 0.99522)
PVA-PE-TF V	Q = 0.3861t+10.7951 (R^2 =0.86639)	Q = 40.69724*(1 - exp(-0.03721*t))	Q = 4.26853t1/2 + 2.11005 (R^2 = 0.99189)	Q = 6.54247t0.41056 (R^2 = 0.9991)

		(R^2 = 0.97071)		

R^2: correlation coefficient; n: model parameter.

Table S2. The fitting date of EO release obtained with different mathematical models at pH = 7.

Sample	Mathematical Model (pH = 7)			
	**Zero order**	**First order**	**Higuchi**	**Peppas**
PVA-PE	Q = 0.15004t + 4.99168(R^2 = 0.78114)	Q = 15.97226*(1 - exp(-0.04664*t)) (R^2 = 0.99565)	Q = 1.71544t1/2 + 1.27059 (R^2 = 0.95646)	Q = 3.30606t0.36145 (R^2 = 0.97964)
PVA-PE-TF I	Q = 0.11909t + 2.87987 (R^2 = 0.85768)	Q = 12.37629*(1 - exp(-0.03404*t)) (R^2 = 0.9989)	Q = 1.31637t1/2 + 0.20215 (R^2 = 0.98168)	Q = 1.61441t0.45475 (R^2 = 0.9842)
PVA-PE-TF II	Q = 0.11553t + 3.7949 (R^2 = 0.78981)	Q = 12.29843*(1 - exp(-0.04561*t)) (R^2 = 0.99717)	Q = 1.31751t1/2 + 0.95029 (R^2 = 0.96213)	Q = 2.49587t0.36513 (R^2 = 0.98377)
PVA-PE-TF III	Q = 0.13951t + 4.47608 (R^2 = 0.79425)	Q = 14.73108*(1 - exp(-0.04543*t)) (R^2 = 0.9942)	Q = 1.5865t1/2 + 1.06818 (R^2 = 0.96215)	Q = 2.91218t0.37147 (R^2 = 0.98173)
PVA-PE-TF IV	Q = 0.14504t + 6.01407 (R^2 = 0.72016)	Q = 16.14138*(1 - exp(-0.05873*t)) (R^2 = 0.99561)	Q = 1.70277t1/2 + 2.14517 (R^2 = 0.92979)	Q = 4.54965t0.29562 (R^2 = 0.98422)
PVA-PE-TF V	Q = 0.15454t + 6.33037 (R^2 = 0.70374)	Q = 17.18749*(1 - exp(-0.05786*t)) (R^2 = 0.99826)	Q = 1.82109t1/2 + 2.1666 (R^2 = 0.91541)	Q = 4.69963t0.30228 (R^2 = 0.96724)

R^2: correlation coefficient; n: model parameter.

Table S3. The fitting date of EO release obtained with different mathematical models at pH = 9.

Sample	Mathematical Model (pH = 9)			
	**Zero order**	**First order**	**Higuchi**	**Peppas**
PVA-PE	Q = 0.19334t + 16.87649 (R^2 = 0.41381)	Q = 28.88448*(1 - exp(-0.20584*t)) (R^2 = 0.98878)	Q = 2.57056$t^{1/2}$ + 9.88311 (R^2 = 0.68523)	Q = 21.48983$t^{0.07468}$ (R^2 = 0.9993)
PVA-PE-TF I	Q = 0.29235t + 10.56505 (R^2 = 0.7854)	Q = 31.51516*(1 - exp(-0.05039*t)) (R^2 = 0.0.98393)	Q = 3.34741$t^{1/2}$ + 3.28457 (R^2 = 0.96451)	Q = 7.44296$t^{0.33315}$ (R^2 = 0.99687)

PVA-PE-TF II	$Q = 0.45789t + 11.44223$ ($R^2 = 0.87564$)	$Q = 47.99125*(1 - exp(-0.03391*t))$ ($R^2 = 0.99012$)	$Q = 5.03962t^{1/2} + 1.27997$ ($R^2 = 0.99357$)	$Q = 6.50089t^{0.44554}$ ($R^2 = 0.99657$)
PVA-PE-TF III	$Q = 0.53616t + 18.42355$ ($R^2 = 0.79442$)	$Q = 57.50489*(1 - exp(-0.04717*t))$ ($R^2 = 0.98738$)	$Q = 6.11337t^{1/2} + 5.22803$ ($R^2 = 0.96744$)	$Q = 12.53061t^{0.34961}$ ($R^2 = 0.99342$)
PVA-PE-TF IV	$Q = 0.47011t + 19.29776$ ($R^2 = 0.72799$)	$Q = 52.28732*(1 - exp(-0.05726*t))$ ($R^2 = 0.99122$)	$Q = 5.50386t^{1/2} + 6.85119$ ($R^2 = 0.93468$)	$Q = 14.53985t^{0.29801}$ ($R^2 = 0.98703$)
PVA-PE-TF V	$Q = 0.42397t +1 3.33066$ ($R^2 = 0.84059$)	$Q = 45.12235*(1 - exp(-0.04161*t))$ ($R^2 = 0.95419$)	$Q = 4.73222t^{1/2} + 3.51862$ ($R^2 = 0.98096$)	$Q = 8.70597t^{0.37303}$ ($R^2 = 0.99607$)

R^2: correlation coefficient; n: model parameter.
